# Functional Diversification of Fungal Glutathione Transferases from the Ure2p Class

**DOI:** 10.4061/2011/938308

**Published:** 2011-11-22

**Authors:** Anne Thuillier, Andrew A. Ngadin, Cécile Thion, Patrick Billard, Jean-Pierre Jacquot, Eric Gelhaye, Mélanie Morel

**Affiliations:** ^1^Unité Mixte de Recherches INRA UHP 1136 Interaction Arbres Microorganismes, IFR 110 Ecosystèmes Forestiers, Agroressources, Bioprocédés et Alimentation, Faculté des Sciences et Technologies, Nancy Université BP 70239, 54506 Vandoeuvre-lès-Nancy Cedex, France; ^2^Department of Biological Sciences, Faculty of Science and Technology, Universiti Malaysia Terengganu, 21030 Kuala Terengganu, Malaysia; ^3^Laboratoire des Interactions Microorganismes-Minéraux-Matière Organique dans les Sols, UMR 7137 CNRS—UHP, Faculté des Sciences et Technologies, Nancy Université BP 70239, 54506 Vandoeuvre-lès-Nancy Cedex, France

## Abstract

The glutathione-S-transferase (GST) proteins represent an extended family involved in detoxification processes. They are divided into various classes with high diversity in various organisms. The Ure2p class is especially expanded in saprophytic fungi compared to other fungi. This class is subdivided into two subclasses named Ure2pA and Ure2pB, which have rapidly diversified among fungal phyla. We have focused our analysis on *Basidiomycetes* and used *Phanerochaete chrysosporium* as a model to correlate the sequence diversity with the functional diversity of these glutathione transferases. The results show that among the nine isoforms found in *P. chrysosporium*, two belonging to Ure2pA subclass are exclusively expressed at the transcriptional level in presence of polycyclic aromatic compounds. Moreover, we have highlighted differential catalytic activities and substrate specificities between Ure2pA and Ure2pB isoforms. This diversity of sequence and function suggests that fungal Ure2p sequences have evolved rapidly in response to environmental constraints.

## 1. Introduction

The glutathione-S-transferase (GST) proteins represent an extended family with high diversity depending on the organism [1–3]. These enzymes are usually able to conjugate glutathione (GSH) to hydrophobic molecules and thus are involved in biotransformation pathways. The addition of GSH onto molecules, previously oxidized by detoxification phase I enzymes, results in the formation of compounds which are usually less toxic and more soluble. These less-toxic peptide derivatives are ready to be excreted or stored. Additionally, these proteins can exhibit other activities such as thiol transferase or peroxidase activities [4–6], suggesting that they could directly participate in the cellular response to oxidative stress. In a recent paper, we have studied the diversity of fungal GSTs, considering zygo-, asco- and basidiomycetes [7]. The major result of this study is that it suggests a link between the number of GST encoding sequences in the analyzed genomes and the fungal way of life. Indeed, 46 and 27 GST-encoding genes have been found in the genomes of *Postia placenta* and *Phanerochaete chrysosporium*, respectively, compared to only 10 for the pathogen *Ustilago maydis* or 11 for the yeast *Saccharomyces cerevisiae. P. placenta *and* P. chrysosporium *are ligninolytic basidiomycetes, which use unspecific oxidative reactions to degrade lignin and, by extension, recalcitrant compounds. In *P. chrysosporium*, extracellular peroxidases are involved in coordination with numerous oxidases [8]. In contrast, *P. placenta *is thought to degrade lignin by secreting various small iron-binding molecules initiating Fenton reactions [9]. Beside these extracellular systems, oxidation of recalcitrant molecules could also occur from the action of various cell-wall linked oxidases such as cytochrome P450 monooxygenases [10]. In the genome of both these fungi, a huge number of cytochrome P450 monooxygenase-related genes have been detected in comparison with other fungi [11, 12]. Such a diversity of cytochrome P450 monooxygenases can be related with the high occurrence of GST encoding genes in their genome and thus to their ability to metabolize recalcitrant compounds found in their natural ecosystem.

Another interesting point is the overrepresentation of a particular class of GSTs called Ure2p both in the genome of *P. placenta* and *P. chrysosporium. *For these species, the Ure2p class represents one-third of the total identified GSTs (9/27 for *P. chrysosporium* and 17/46 for *P. placenta*). *P. chrysosporium* Ure2p sequences cluster into two different subclasses. The first one, enclosing 8 of the 9 sequences, is related to the single Ure2p isoform of *S. cerevisiae*. In yeast this protein is known to act as a negative regulator of the nitrogen catabolite regulation (NCR) in response to primary nitrogen source by disabling Gln3p to activate transcription [13]. Moreover, the inability of a *URE2 *deleted mutant strain (Δ*ure2*) to grow in presence of hydrogen peroxide (H_2_O_2_) has been demonstrated, and a relationship between diminishing levels of GSH and peroxide sensitivity was established. It was suggested that the susceptibility of the Δ*ure2* strain to the exogenous H_2_O_2 _ can result from increased GSH degradation due to the deregulated localization of the gamma-glutamyl transpeptidase activating factors Gln3p/Gat1p [14]. The last *P. chrysosporium* sequence, named PcUre2p1, clusters in another group containing *GSTA* (AN4905) from *Aspergillus nidulans*. *GSTA* contributes to metal detoxification and contrary to the yeast isoform, it is not involved in the NCR [15]. 

In this study, we performed an exhaustive analysis of Ure2p sequences from fungal genomes, focusing on *P. chrysosporium* to correlate the diversity of sequences with the diversity of functions.

## 2. Materials and Methods

### 2.1. Strains and Culture Conditions

The strain of *P. chrysosporium* used is the homocaryon RP-78. Its genome has been fully sequenced and annotated [16]. The fungus was maintained on 4% malt, 3% agar plates. The sporulation conditions have been described previously [17]. The liquid culture medium consists of 5 mM sodium acetate pH 4.5, 1% glucose, 1 mM ammonium tartrate, 1% (v/v) base medium (20 g/L KH_2_PO_4_, 5 g/L MgSO_4_, 1 g/L CaCl_2_), 7% (v/v) trace medium (1.5 g/L Nitrilotriacetate, 3 g/L MgSO_4_, 1 g/L NaCl, 0.1 g/L FeSO_4_·7H_2_O, 0.1 g/L CoCl_2_, 0.1 g/L ZnSO_4_·7H_2_O, 0.1 g/L CuSO_4_, 10 mg/L AlK(SO_4_)·12H_2_O, 10 mg/L H_3_BO_3_, 10 mg/L NaMoO_4_·2H_2_O) and 225 *μ*M MnCl_2_. The fungal inoculation was made by adding 2.5·10^6^ spores (OD_650_ = 0.5) per flask containing 100 mL of liquid medium. For the cultures on wood, the fungus was first grown for 3 days on malt agar plates, and autoclaved wood chips were then placed on the fungal mat. The fungus was harvested from the wood chips 15 days later. The fungus was also grown in liquid medium containing polycyclic aromatic hydrocarbons (PAH). For this condition, the fungal pellets were first grown without PAH for 5 days to yield biomass. The fungus was then transferred into new flasks containing PAH. To prepare these flasks, a stock solution of PAH was solubilised in hexane and added to the flasks to reach final quantities of 2.25 mg phenanthrene, 0.225 mg fluorene, 0.225 mg fluoranthene, and 0.225 mg anthracene per flask. 100 mL of liquid culture medium, the composition of which is described above but without glucose, was added in each flask after complete evaporation of hexane. 

### 2.2. Sequence Analysis

For the global phylogenetic analysis, 189 sequences from both *Ascomycetes* and *Basidiomycetes* were found in NCBI genome database (http://www.ncbi.nlm.nih.gov/genome/) using blastp with all *P. chrysosporium* Ure2p sequences as template. The deeper analysis focusing on *Basidiomycetes* was performed using data from the Fungal Genomics Program of the Joint Genome Institute (JGI) (http://genome.jgi-psf.org/Phchr1/Phchr1.home.html) using blastp with *P. chrysosporium* Ure2p sequences as template. 153 basidiomycete sequences were retrieved from this database. Phylogenetic and molecular evolutionary analyses were conducted using *MEGA* version 5 [18]. Alignments were done with Muscle and the phylogenetic tree was constructed with the neighbour-joining method. Predictions of subcellular localization were done using WolfpSort (http://wolfpsort.org/) and Mitoprot (http://ihg2.helmholtz-muenchen.de/ihg/mitoprot.html) softwares. Synteny was determined through the Fungal Genomics Program of the Joint Genome Institute (JGI). All references of the sequences used in this study are given in additional data. 

### 2.3. Gene Expression

Gene expression was checked by semiquantitative Reverse Transcription Polymerase Chain Reaction (RT-PCR). Total RNA isolation was performed using the RNeasy Plant Mini Kit (Qiagen, Hilden, Germany). RNase-free DNase treatment (Qiagen) was applied according to the manufacturer's protocol to avoid genomic DNA contamination. Reverse transcription (RT) reactions were performed with 500 ng of total RNA using the Masterscript Kit (Prime) according to the manufacturer's protocol. RT products were amplified by PCR in the following conditions: DNA denaturation for 1 min at 95°C and 33 cycles at 95°C for 5 s, 52°C for 45 s, and 72°C for 1 min using Go Taq DNA polymerase (Promega). The suitability of the extracted RNA for RT-PCR amplification was checked by performing RT-PCR control experiments in the same amplification conditions with the ubiquitin encoding gene. 

### 2.4. PAH Quantification

PAHs from total culture media were successively extracted three times with dichloromethane (DCM) (v/v) in separation funnels. DCM extracts were evaporated in a speedVac concentrator (Thermo Scientific RC1010), and dried samples were dissolved in acetonitrile for HPLC analyses using a Dionex UltiMate 3000 system with a 5 *μ*m Agilent Eclipse PAH of 4.6 × 150 mm C18 reverse phase column maintained at 30°C. The compounds were detected and identified through Dionex UV photodiode array detector at 254 nm. The elution solution of 70% acetonitrile and 30% H_2_O was used with a flow rate of 2 mL/min.

### 2.5. Microscopic Analysis

*P. chrysosporium* pellets grown in liquid cultures with or without PAH as indicated below were crushed, and observations were made with an epifluorescence microscope (Nikon E600) with an HQ-FITC-BP filter cube (Chroma) for excitation at 345 nm and emission at 485 nm (DAPI). Pictures were collected with a Nikon D60 Digital SLR camera.

### 2.6. Production and Purification of Recombinant Proteins

Amplifications of GST cDNAs were performed from RT products obtained as described above, using the high proof Herculase DNA polymerase (Stratagene). The PCR products were cloned into the NcoI and BamHI sites of the pET-3d vector (Novagen) resulting in a construction devoid of a His-Tag. The recombinant plasmid was then used to transform *Escherichia coli* strain BL21 (DE3) cotransformed by the helper plasmid pSBET in order to provide the rare t-RNAs for AGG and AGA codons [19]. After induction with isopropyl *β*-D-1-thiogalactopyranoside (IPTG), proteins were purified using a combination of gel filtration and anion exchange chromatography as described in Rouhier et al. [20].

### 2.7. Enzymatic Activity Measurements

The activities of the recombinant proteins were assayed spectrophotometrically in 1 mL reaction medium using 8.5 *μ*M PcUre2p4, 1.3 *μ*M PcUre2p6, and 0.55 **μ**M PcUre2p1. GST activity was assayed in 50 mM phosphate buffer pH 6.5, 2 mM chlorodinitrobenzene (CDNB) and 0.2 to 12.5 mM GSH. The pH dependency of PcUre2p4 and PcUre2p6 activities was assessed in 50 mM phosphate buffer pH 5.8 to pH 8.0 using 4 mM CDNB and 6 mM GSH. The other activities were assayed in 30 mM Tris-HCl pH 8, 1 mM EDTA buffer, 1 mM *β*-hydroxyethyl disulphide (HED), dehydroascorbate (DHA) or peroxides as substrates, 0.2 to 12.5 mM GSH, 180 *μ*M NADPH, and 0.5 IU of purified glutathione reductase [21–23]. Oxidation of NADPH and transformation of CDNB were both followed at 340 nm. 

## 3. Results 

### 3.1. Phylogenetic Analysis

Ure2p sequences have been identified within available genome database from NCBI using blastp and PcUre2p amino acid sequences as template. 

Concerning bacterial genomes, PcUre2p homologues have been identified only in *Actinobacteria*, *Cyanobacteria,* and *Firmicutes* phyla. No similar sequence was found for *Archaea* while some were identified for *Spirochaetales* and *Proteobacteria* except the epsilon subdivision. Among eukaryotic organisms, only *Stramenopiles*, *Dictyosteliida,* and fungi possess similar sequences.

**Figure 1:**
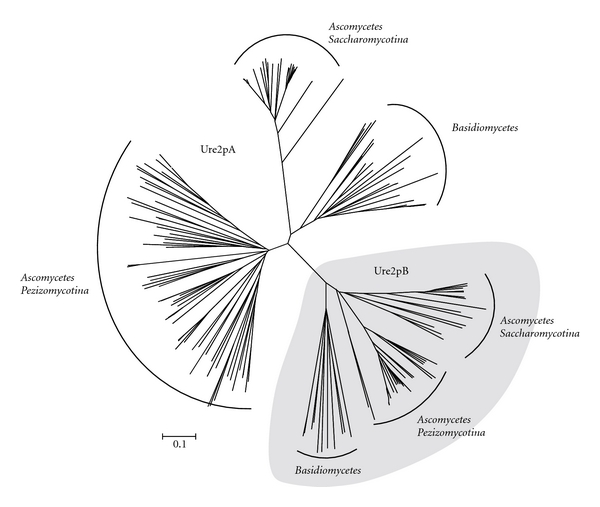
Neighbour joining reconstruction of the Ure2p protein sequences from *Ascomycetes* and *Basidiomycetes* NCBI genome database. Species names and sequence references are given in additional data. The bar represents a distance scale of 0.1 mutations per site.

**Figure 2:**
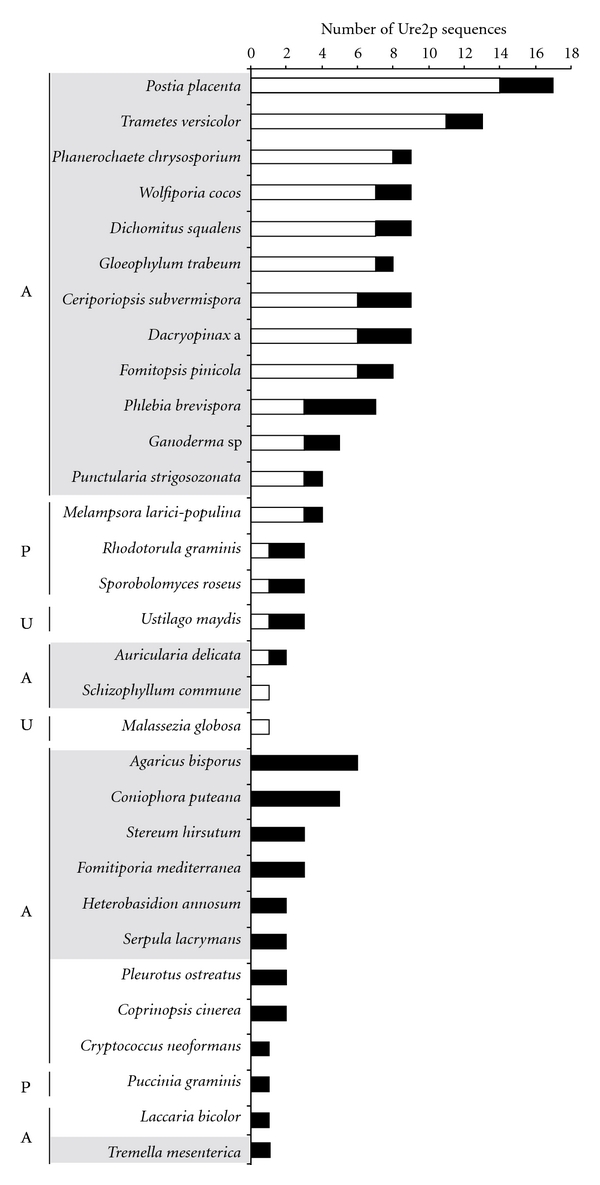
Number of Ure2p sequences found in all available genomes of *Agaricomycotina* (A), *Pucciniomycotina* (P), and *Ustilaginomycotina * (U) from the JGI Fungal genomics Program. A preliminary phylogenetic analysis allowed to differentiate Ure2pA (white bars) from Ure2pB sequences (black bars). Wood-decaying fungi are framed in grey.

Ure2p amino acid sequences from *Ascomycete* and *Basidiomycete* genomes have thus been collected and analyzed through a phylogenetic approach (Figure 1). All sequence references are given in additional data. Two main subclasses, previously called Ure2p and cluster 2 [2, 7], are distinguishable and have been renamed Ure2pA and Ure2pB, respectively. Both subclasses are present in all fungal phyla. These sequences were grouped according to the fungal taxonomy, suggesting a recent diversification of Ure2p sequences among each phylum. While Ure2pB sequences from *Saccharomycotina* and *Pezizomycotina* are close to each other, *Saccharomycotina* sequences are rather related to *Basidiomycotina*. We then focused our analysis on *Basidiomycotina* by performing an exhaustive analysis of Ure2p sequences in all available basidiomycete genomes from the JGI Fungal Genomics Program. Ure2p sequences have been found in all the species tested. A phylogenetic analysis of the sequences allowed classifying them in the Ure2pA or Ure2pB subclasses (data not shown). The numbers of sequences from each cluster are reported in Figure 2. Two species (*Malassezia globosa *and* Schizophyllum commune*) possess only one sequence from the Ure2pA subclass, some other (*Agaricus bisporus, Coniophora puteana, Stereum hirsutum, Fomitiporia mediterranea, Heterobasidion annosum, Serpula lacrymans, Pleurotus ostreatus, Coprinopsis cinerea, Cryptococcus neoformans, Puccinia graminis*, *Laccaria bicolor, *and* Tremella mesenterica*) possess only sequences from Ure2pB, while the other fungi exhibit both isoforms. There is no relationship between this observation and the taxonomy of the fungi. Globally, the fungi exhibiting the highest number of sequences are wood decaying fungi (Figure 2, grey shadowing), however some species also involved in wood degradation show a relatively small number of Ure2p sequences (*Auricularia delicata, Schizophyllum commune, Heterobasidion annosum, Serpula lacrymans, Pleurotus ostreatus, and Tremella mesenterica*). Thus, the diversification of Ure2p sequences does not seem to be related with fungal evolution, nor with the wood degrading properties of the fungi, suggesting the occurrence of other environmental pressures. 

### 3.2. Synteny

**Figure 3:**
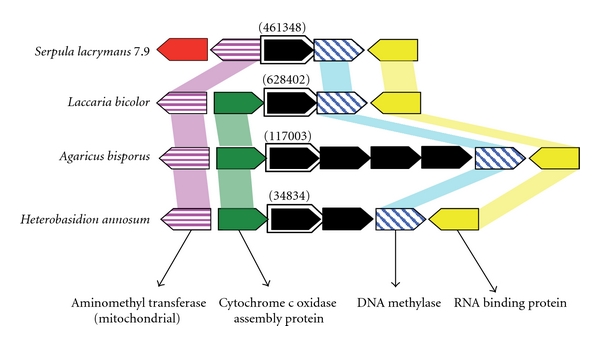
Conserved synteny of Ure2pB sequences among 4 *Agaricomycotina* species (*S. lacrymans, L. bicolor, A. bisporus, *and *H. annosum*). Ure2p genes are in black. Protein IDs of the sequences are reported onto the figure into brackets. The putative function of the genes surrounding Ure2p was obtained by sequence homology using blastp.

Globally the Ure2p synteny is not conserved among *Agaricomycotina*, except for Ure2pB sequences in few species which possess only these isoforms (*S. lacrymans*, *L. bicolor,* and *H. annosum*) (Figure 3). Ure2pB genes are surrounded with putative mitochondrial aminomethyltransferase, cytochrome c oxidase assembly protein, DNA methylase and RNA binding protein. However the putative link between Ure2pB and either mitochondrial metabolism or nucleic acid modification pathways has not been documented. 

### 3.3. Expression of PcUre2p Genes in *P. chrysosporium*

**Figure 4:**
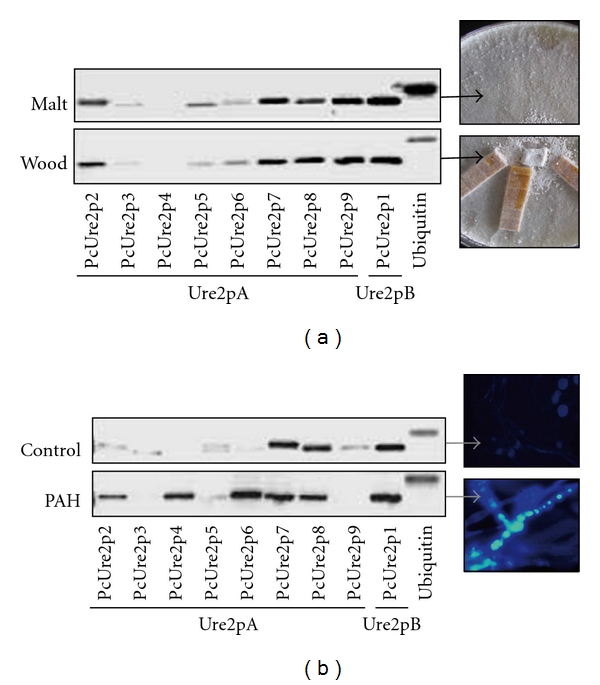
Gene expression of *P. chrysosporium* Ure2p measured by semiquantitative RT-PCR. (a) Ligninolytic condition (wood) versus nonligninolytic condition (Malt). (b) PAH treatment for 10 days; in this condition, PAH are internalized inside lipid vesicles (see photo) versus a control without PAH. The ubiquitin coding gene was amplified as a control. Two biological repetitions have been performed.

The expression of all *P. chrysosporium* Ure2p coding genes was monitored in two conditions using semiquantitative RT-PCR (Figure 4). The first one corresponds to ligninolytic conditions (Figure 4(a)), and the second is a condition where the fungus is exposed to genotoxic PAHs [24]. In this condition, *P. chrysosporium* was able to dissipate between 15 to 30% of the initially added PAH in the culture medium after 10 days of incubation, the highest removal occurring with fluorene and fluoranthene. Taking advantage of the fluorescent properties of PAH, we were able to detect their intracellular accumulation in *P. chrysosporium* in our culture conditions after 10 days of treatment (Figure 4(b)). This accumulation occurred both in hyphae and spores, not in vacuoles but rather in lipid vesicles as shown before in *Fusarium solani* [25].

No difference was highlighted concerning gene expression in ligninolytic (wood) compared to nonligninolytic (malt) conditions (Figure 4(a)): PcURE2p4 was not expressed, PcURE2p3, PcURE2p5, and PcURE2p6 were slightly expressed, while the others were constitutively expressed in both conditions. 

PcURE2p1, PcURE2p7, and PcURE2p8 were constitutively expressed with and without PAH treatment, while the PcURE2p4 and PcURE2p6 genes were specifically induced after PAH treatment (Figure 4(b)). PcURE2p4, PcURE2p6, and PcURE2p8 genes are repeated in tandem but are not regulated in the same manner suggesting a different regulatory system between PcURE2p8 and the two others. PcURE2p2, PcURE2p3, PcURE2p5, and PcURE2p9 are not or very slightly expressed in both conditions. 

### 3.4. Analysis of Ure2p Sequences

**Figure 5:**
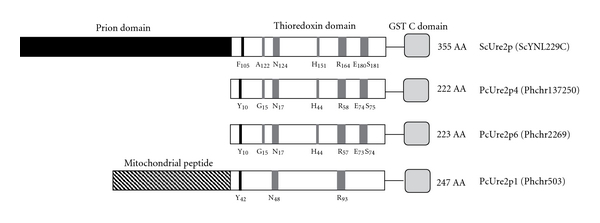
Comparative analysis of the Ure2p sequence from *Saccharomyces cerevisiae* and Ure2p1, Ure2p4 and Ure2p6 from *Phanerochaete chrysosporium*. Only the amino acids of the N-terminal domain known to be involved in the activity of the proteins have been reported. The protein IDs are given into brackets.

The amino acid sequences of PcUre2p4 and PcUre2p6 are very similar to one another showing 83.2% identity [7]. PcUre2p1 exhibits 26% and 29% of identity with PcUre2p4 and PcUre2p6, respectively. The proteins have the classical organization of GST, that is, a GSH-binding domain (Thioredoxin domain or G-site) and a *α*-helical domain (GST C-domain) (Figure 5). The Trx-domain of these proteins is highly conserved, exhibiting amino acids known to be involved in the interaction with GSH in the *S. cerevisiae* isoform (ScUre2p) [26, 27]. Asn124 and Arg164 (ScUre2p numbering) are shared by ScUre2p, PcUre2p1, PcUre2p4, and PcUre2p6. Glu180 and Ser181 residues are only conserved in ScUre2p, PcUre2p4, PcUre2p6 but not in PcUre2p1. Ala122 is replaced by Gly in PcUre2p4 and PcUre2p6. Moreover, the three *P. chrysosporium* isoforms exhibit a Tyr instead of Phe105. Another interesting point is the presence of a putative mitochondrial targeting peptide in PcUre2p1, suggesting a potential role for this protein in the organelle. The major difference between the yeast isoform and the other fungal sequences is the presence of the prion domain in ScUre2p [28]. This Gln/Asn rich sequence at the N-terminal end is required for aggregation properties of the protein. However, deletion of this N-terminal region has no effect on the stability or folding of the protein *in vitro* [29]. Among all the fungi analysed, only sequences from *Saccharomycotina* exhibit the N-terminal prion domain, suggesting that the other proteins have evolved separately in the other fungi, the most likely hypothesis being the acquisition of this property in *Saccharomycotina*. 

### 3.5. Biochemical Properties of PcUre2p1, PcUre2p4, and PcUre2p6

**Figure 6:**
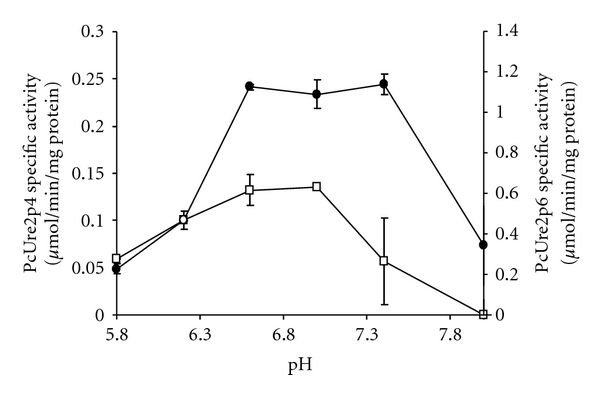
pH dependency of PcUre2p4 (□) and PcUre2p6 (•) activities using CDNB as substrate. Experimental details are given in Section 2.

The differences in the amino acid sequences are likely to influence the catalytic properties of the corresponding proteins. To test this hypothesis, the recombinant PcUre2p1, PcUre2p4, and PcUre2p6 proteins have been produced in *Escherichia coli* and purified. Their activities have been tested *in vitro *using various substrates (Table 1). PcUre2p4 and PcUre2p6 exhibited activity against CDNB, suggesting classical GST activity. For both enzymes, the highest specific activity was measured around pH 6.5 (Figure 6). The affinity of PcUre2p4 and PcUre2p6 are, respectively, 2.62 mM ± 0.26 and 3.12 mM ± 0.20 for GSH, and 2.46 mM ± 0.62 and 2.49 mM ± 0.71 for CDNB. 

PcUre2p4 has been found to be inactive as thiol transferases and dehydroascorbate reductases. Contrary to ScUre2p, PcUre2p4 and PcUre2p6 had no GSH peroxidase activity whatever the peroxide used (hydrogen peroxide, t-butyl peroxide, or cumene peroxide). The purified recombinant PcUre2p1 was not active with both CDNB and peroxides but did reduce HED and DHA, thus exhibiting thioltransferase activity. 

## 4. Discussion 

While the single Ure2p isoform of *S. cerevisiae* has been well studied for its prion properties, its involvement in the NCR, and its role in the oxidative stress response [13, 14, 28, 29], this study is the first description of Ure2p in other fungi. This class is extended in basidiomycetes and especially in saprophytic fungi, suggesting a putative link between these GSTs and the degradative properties of the fungi. In many species, Ure2p genes are duplicated in tandem, revealing a monophyletic phylogenetic relationship between genes as described recently for insect GSTs [32]. Moreover, a tyrosine hydroxyl group thought to act as a hydrogen bond donor to the sulphur of GSH thus lowering its pK_a_ to stabilize a nucleophilic thiolate [33] is conserved in the first 15 amino acid residues of fungal sequences. The presence of this residue at the end of the first *β*-sheet defines a subgroup of GSTs called Y-GST type [34]. Based on the taxonomic distribution, it has been suggested that this type has evolved more recently compared to the so-called S/C-GST type found in other GST classes such as Omega [35]. 

*P. chrysosporium* exhibits nine Ure2p isoforms. We have tried here to decipher the significance of this diversification and the likely physiological roles of these enzymes. PcUre2p4 and PcUre2p6 from the Ure2pA subclass exhibit glutathionylation activity with CDNB, while PcUre2p1 from the Ure2pB subclass possesses only deglutathionylation (also called thioltransferase) activity. Incidentally, ScUre2p, which clusters into the Ure2pA subclass possesses deglutathionylation and peroxidase activity. However, none of the tested PcUre2p exhibits the peroxidase activity described in yeast, suggesting other functions in cell. To test this hypothesis, complementation tests using a *S. cerevisiae *ΔUre2p mutant have been performed (data not shown). The results showed that PcUre2p1, PcUre2p4, and PcUre2p6 are not able to restore the phenotype of the mutant concerning both the sensitivity to H_2_O_2_ [14] and its function in the NCR. However, a homologue of the yeast Gln3p is present in the genome of *P. chrysosporium* (Phchr 43861), suggesting that NCR could exist in this fungus. The main conclusion is that PcUre2p1, 4, and 6 do not have the same activity as ScUre2p in protecting cells against oxidative, heavy metal, or aromatic compounds stress, even if PcUre2p4 and PcUre2p6 belong to the same subclass as the yeast isoform. We can hypothesize that PcUre2p proteins are not directly involved in rescuing oxidative stress such as GSTs exhibiting peroxidase activities, but rather acts to detoxify specific substrates. The specific expression of PcUre2p4 and PcUre2p6 genes after PAH treatment is in accordance with the enzymatic data. PAHs are aromatic molecules that are degraded by at least three mechanisms in fungi: one uses the cytochrome P-450 system which is composed of a superfamily of monooxygenases, one uses the Fenton reaction [36], and the other uses the soluble extracellular enzymes of lignin catabolism, including lignin peroxidase (LiP), manganese peroxidase (MnP), and laccases which are nonspecific and oxidize a wide variety of organic compounds [37, 38]. However, in our conditions, no LiP or MnP activity was detected in the culture medium; we rather observed an intracellular storage of PAH in lipid vesicles as previously shown in *Fusarium solani* [25]. We can hypothesize a role of PcUre2p4 and 6 in intracellular PAH glutathionylation, transport, or oxidative stress rescue. PcUre2p1 gene was constitutively expressed in our conditions. In *A. nidulans*, the homologue *AnGSTA* contributes to metal and xenobiotic detoxification, as evidenced by the sensitivity to selenium, silver, nickel, sulphanilamide, and pyrrolnitrin of strains lacking a functional copy of *GSTA* [15]. The analysis of the soluble proteome of *P. chrysosporium* has revealed the presence of PcUre2p1 in a standard culture condition with a relative low abundance [39]. Moreover, the authors have shown a 3-fold upregulation of the protein after copper treatment. Another study revealed an induction of PcUre2p1 gene in response to nonylphenol [40]. Because of its putative mitochondrial localization, the protein may have a role in reducing toxic molecules in this organelle. A bacterial homolog of PcUre2p1 called YfcG with 43% amino acid identity has been characterized in *Escherichia coli* [41]. Since this Ure2pB isoform is largely represented among fungi (Figure 2), the question of a putative bacterial origin is open.

In conclusion, we showed in this study that Ure2p GSTs in *P. chrysosporium* do not have the same role as ScUre2p. The repartition into 2 distinct subclasses in many fungi suggests that these proteins have evolved separately inside the Ure2p class, adapting their functional specificities to environmental constraints. It is indeed assumed that the genes that are under external pressure may evolve to carry out myriad functions with diverse substrate specificities through local duplications followed by diversification. The putative link between the saprophytic properties of the fungi and the function of the Ure2p proteins remains to be elucidated to consider the use of these GSTs with high substrate specificities as environmental biomarkers.

## Figures and Tables

**Table 1:** Specific activities of recombinant *P. chrysosporium* and yeast proteins, using CDNB, HED, DHA, and peroxides (hydrogen peroxide, ter-butyl hydroperoxide, and cumene hydroperoxide). The specific activities are expressed in *μ*mol/min/mg protein.

	CDNB	HED	DHA	Peroxides
ScUre2p	—	Active*	Active*	Active**
PcUre2p4	0.18 ± 0.01	—	—	—
PcUre2p6	1.21 ± 0.03	2.82 ± 0.08	0.76 ± 0.03	—
PcUre2p1	—	4.07 ± 0.88	2.90 ± 0.09	—

The specific activities concerning ScUre2p are not reported since the experimental procedures were different from the one used in this study.

*according to Zhang and Perrett [30], **according to Bai et al. [31].
